# Epidemiology of Neuralgic Amyotrophy—A Retrospective Analysis of Data From a Large German Health Insurance Company

**DOI:** 10.1002/mus.70059

**Published:** 2025-11-14

**Authors:** Johannes Fabian Holle, Andreas Leha, Carolin Polte, Volker Limmroth, Wolfram Windisch, Maximilian Zimmermann

**Affiliations:** ^1^ Department of Neurology Cologne‐Merheim, Hospitals of the City of Cologne Cologne Germany; ^2^ Health Faculty/Department for Human Medicine University of Witten/Herdecke Witten Germany; ^3^ Department of Medical Statistics University Medical Center Göttingen Göttingen Germany; ^4^ AOK Federal Association Outpatient Analysis and Care Scientific Institute of the AOK Berlin Germany; ^5^ Cologne‐Merheim Lung Clinic, Hospitals of the City of Cologne Cologne Germany

**Keywords:** epidemiology, incidence, neuralgic amyotrophy, phrenic neuropathy, prevalence

## Abstract

**Introduction/Aims:**

Neuralgic amyotrophy (NA, Parsonage–Turner syndrome) is a common, multifocal, autoimmune inflammatory disease that predominantly affects proximal nerve segments of the shoulder girdle. Despite the high incidence of 100/100,000, epidemiologic data based on larger cohorts are still lacking. This study aims to address this issue.

**Methods:**

A retrospective evaluation of billing data from an average of approximately 26,000,000 insured persons of a large German health insurance company was performed from 2013 to 2022. In addition to descriptive statistical methods, regression analyses were carried out.

**Results:**

The incidence and prevalence of NA were 7.7–12.8 and 19.7–21.6 per 100,000 people, respectively. During the study period, a steady decline in incidence and, to a lesser extent, prevalence was observed. The diagnosis was made significantly more frequently in the first quarter of the year than in any other quarter. The prevalence of a simultaneously coded diaphragmatic paresis (indicating the involvement of the phrenic nerve) was 0.45%.

**Discussion:**

Compared with prospectively collected data, the data in this study revealed an approximately 90% lower incidence of NA. It is likely that the majority of cases are not correctly diagnosed and are therefore not captured in the billing data. The involvement of the phrenic nerve in patients with NA also appears not to be recognized in most cases. According to the current pathophysiological model of NA, an immunological trigger is necessary to initiate the disease process. Our data support the accuracy of this model.

## 1 Introduction

Neuralgic amyotrophy (NA, Parsonage–Turner syndrome) is a common, underdiagnosed, multifocal autoimmune inflammatory disease that predominantly affects proximal nerve segments of the shoulder girdle.

The classic clinical course begins with pronounced shoulder pain, followed by paresis within hours to days, particularly of the shoulder girdle muscles. In addition to this classic phenotype, approximately one‐third of patients present with paralysis of the distal arm muscles, and up to 10% present with lumbosacral manifestations [1, 2].

The pathophysiology of the disease—as it is currently understood—is based on the combination of a genetic predisposition, an immunological trigger [3] and a mechanical trigger (e.g., prolonged physical activity in the shoulder girdle area) [4]. The latter factor leads to increased permeability of the blood‐nerve barrier via microtraumatization of the nerves, allowing cellular or humoral components of the immune system to reach the endoneural space and triggering an autoimmune‐inflammatory process [5].

Overall, the disease has been found to have a significantly worse outcome and greater economic significance than previously assumed. Up to 25% of employees who develop NA are unable to return to their previous work in the long term; for the Netherlands, with approximately 7.1 million employees, the annual costs caused by lost production have been calculated at USD 40,000,000 [6]. Earlier studies estimated the annual incidence to be between 1 and 3 cases per 100,000 individuals [7, 8]. However, a more recent, prospective study reported a 30 to 100 times higher incidence, approximately 100 cases per 100,000 people [6].

The available epidemiological data is limited, especially data from cohorts with high case numbers. In the German context, in particular, there is a lack of current figures on NA, yet these figures are crucial for well‐informed discussions and the development of needs‐based diagnostic and therapeutic strategies. Therefore, the present study aimed to provide incidence and prevalence figures by analyzing the epidemiological data of a German health insurance fund.

## 2 Methods

There are currently 11 legally independent health insurance funds in Germany under the name ‘Allgemeine Ortskrankenkasse’ (AOK), which together insured approximately 27 million people in 2021. With a market share of approximately 37%, the AOK is Germany's largest health insurance company [9].

Our work was based on the retrospective evaluation of billing data in accordance with § 295 SGB V (refers to billing for services provided by contract physicians) and § 301 SGB V (refers to billing for hospital treatment and services provided by rehabilitation facilities) of AOK insured persons and the KM6 membership statistics from 2013–2022. These statistics are collected once a year and published by the Federal Ministry of Health and the Federal Association of Health Insurance Funds. Persons who were insured with the AOK for at least 1 day in the period mentioned and for whom the ICD‐10 indicator G54.5 (Neuralgic Amyotrophy) was coded as a confirmed diagnosis were selected for the evaluation. Cases that were billed using other ICD‐10 codes, such as M25.51 for joint pain in the shoulder region, were deliberately excluded. German hospitals assign a preliminary diagnosis code when admitting patients, but the final code is not assigned until inpatient treatment is complete. This code is used to bill the statutory health insurance funds. Therefore, we can assume that the correct diagnosis was not made in cases of NA billed under other ICD‐10 codes. Additionally, patients were identified who were coded with both the ICD‐10 indicator G54.5 (neuralgic amyotrophy) and the indicator J98.6 (diaphragmatic paralysis).

The data were provided and analyzed anonymously. The incidence and prevalence rates were calculated with 95% confidence intervals. Where indicated, the percentages are scaled to obtain figures per 100,000. The incidence of NA was compared between the first and other annual quarters via a Poisson regression model that includes year and quarter as predictors. The resulting estimates are presented with 95% confidence intervals and *p*‐values to test the null hypothesis of no association. Normalization to the total number of insured patients per quarter was not possible, as these data were not available. Since the total number of patients is not expected to fluctuate with the quarter, the comparison of the numbers between quarters is considered valid. Logistic regression models were used to test for a trend in the incidence and prevalence of NA over time. The resulting regression coefficients were reported as odds ratios, along with their respective 95% confidence intervals and *p*‐values, to test the null hypothesis of no correlation. We also calculated the prevalence of concomitant diaphragmatic paralysis (J98.6) within the cohort of insured persons diagnosed with NA.

The significance level was set to alpha = 5% for all the statistical tests. All analyses were performed with the statistical software R (version 4.4.0; R Core Team 2024) using the R package emmeans (version 1.10.7; Lenth 2025) for the contrast tests. The graphics were created using Excel for Mac version 16.88 (Microsoft, Redmond, WA, USA).

## 3 Results

From 2013 to 2022, an average of 25,597,787 people were insured with the AOK each year. The median age was 50–59 years (Tables 1 and 2).

**TABLE 1:** Incidence and prevalence during the study period.

	Total insured	Incidence NA (per 100.000)	Prevalence NA (per 100.000)	Prevalence diaphragmatic paresis in NA (%)
2013	24,114,347	12.8 [12.4; 13.3]	21.2 [20.7; 21.8]	0.3 [0.2; 0.5]
2014	24,158,146	12.3 [11.9; 12.8]	21.4 [20.8; 22]	0.3 [0.2; 0.5]
2015	24,302,580	11.8 [11.4; 12.3]	21.6 [21; 22.2]	0.4 [0.2; 0.6]
2016	25,014,139	10.9 [10.5; 11.4]	21.4 [20.8; 22]	0.5 [0.3; 0.7]
2017	25,735,948	10.4 [10; 10.8]	20.9 [20.4; 21.5]	0.4 [0.3; 0.7]
2018	26,2644,75	9.5 [9.2; 9.9]	20.2 [19.7; 20.8]	0.5 [0.4; 0.8]
2019	26,477,052	9.7 [9.4; 10.1]	20.6 [20; 21.1]	0.5 [0.3; 0.7]
2020	26,770,605	8.9 [8.5; 9.2]	20.1 [19.6; 20.7]	0.5 [0.3; 0.7]
2021	26,762,835	8.6 [8.3; 9]	20.4 [19.9; 21]	0.5 [0.4; 0.8]
2022	26,899,648	7.7 [7.4; 8.1]	19.7 [19.2; 20.3]	0.6 [0.4; 0.8]
Mean	25,649,978	10.3 [9.9; 10.7]	20.7 [20.2; 21.3]	0.5 [0.3; 0.7]

**TABLE 2:** Incidence by annual quarter.

	Incidence NA (per 100.000)	Rate ratio compared to quarter 1	*p*
Quarter 1	11.3 [10.9; 11.7]		
Quarter 2	9.7 [9.3; 10.1]	0.86 [0.83; 0.89]	< 0.001
Quarter 3	10.0 [9.6; 10.4]	0.88 [0.85; 0.91]	< 0.001
Quarter 4	9.9 [9.5; 10.3]	0.88 [0.83; 0.89]	< 0.001

**FIGURE 1:**
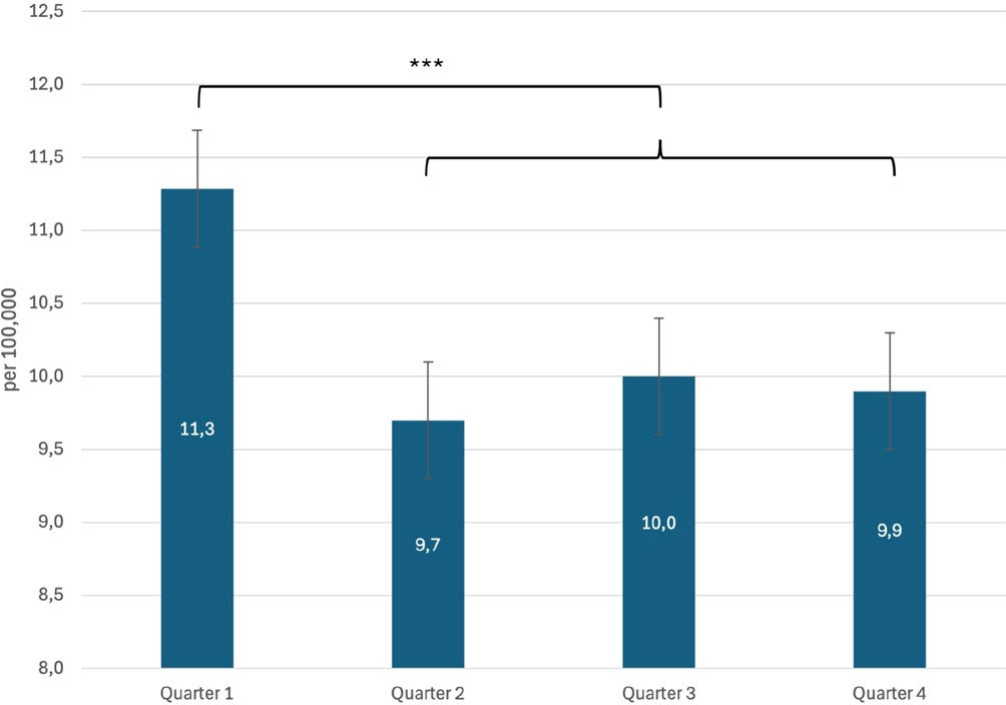
Incidence by annual quarter. The incidence of NA is significantly higher in the first quarter than in quarters two through four. ***p < 0.001.

**FIGURE 2:**
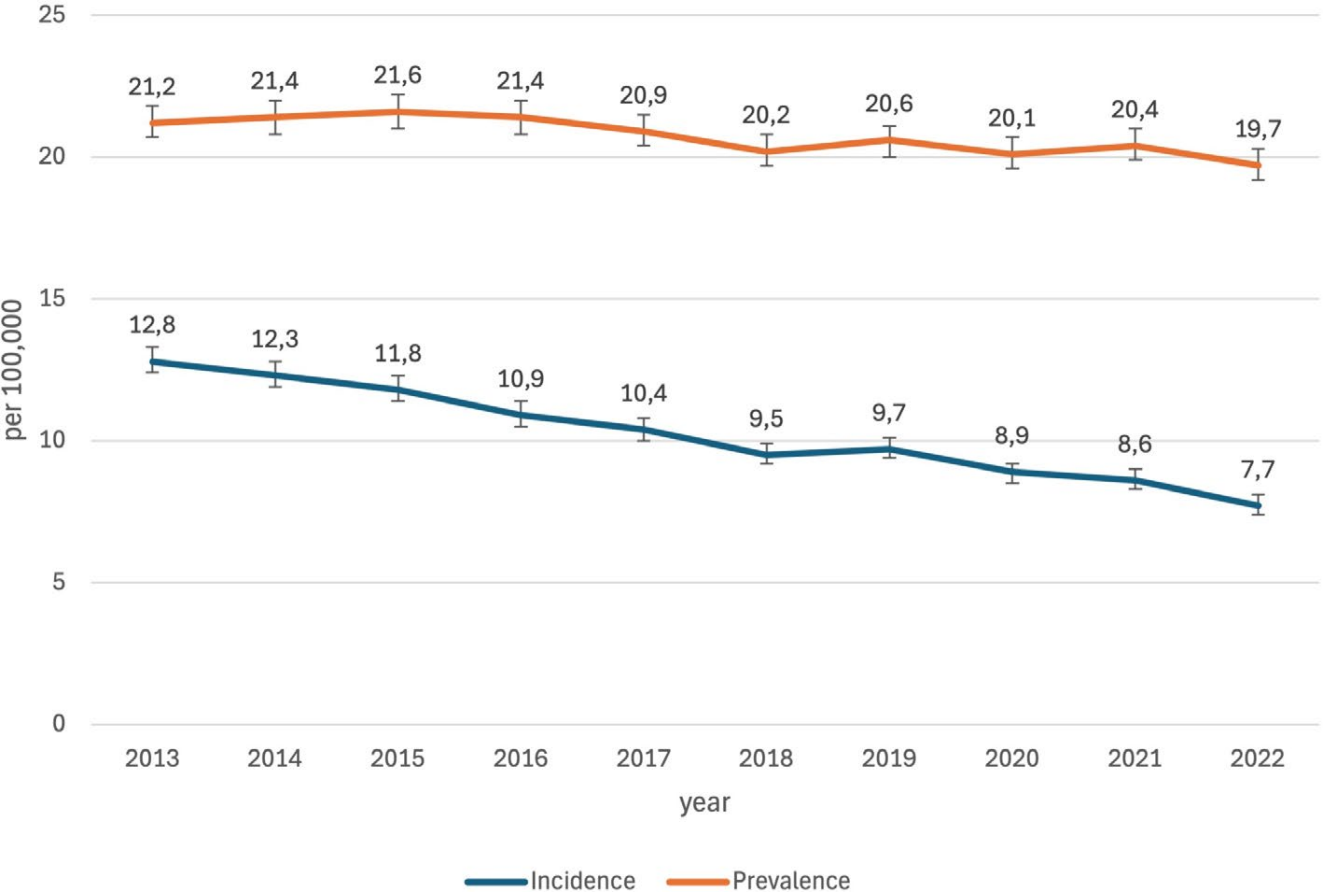
Incidence and prevalence during the study period. During the study period, a steady decline in the incidence and, to a lesser extent, prevalence of NA was observed.

On average, the first diagnosis of NA was made in 2620 insured persons per year, corresponding to an incidence of 10.3/100,000 (Table 1). A more frequent diagnosis was made during the first quarter than during the second, third and fourth quarters (on average, 723 versus 632 cases, corresponding to an extrapolated annual incidence of 11.3 versus 9.9/100,000, as shown in Table 2 and Figure 1). This discrepancy was highly statistically significant (*p* < 0.001). Overall, there was a steady, significant decline in the incidence of NA from 12.8 in 2013 to 7.7/100,000 in 2022 over the time period analyzed (OR per year 0.948; 95% CI [0.944;0.952]; *p* < 0.001) (Figure 2).

**FIGURE 3:**
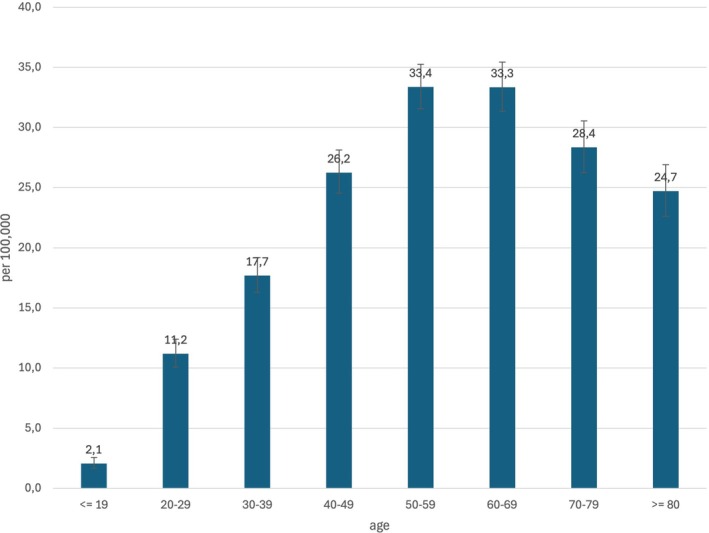
Prevalence in different age groups. The highest prevalence was seen in the 50–59 years age group, and the lowest in the ≤ 19 years age group.

**FIGURE 4:**
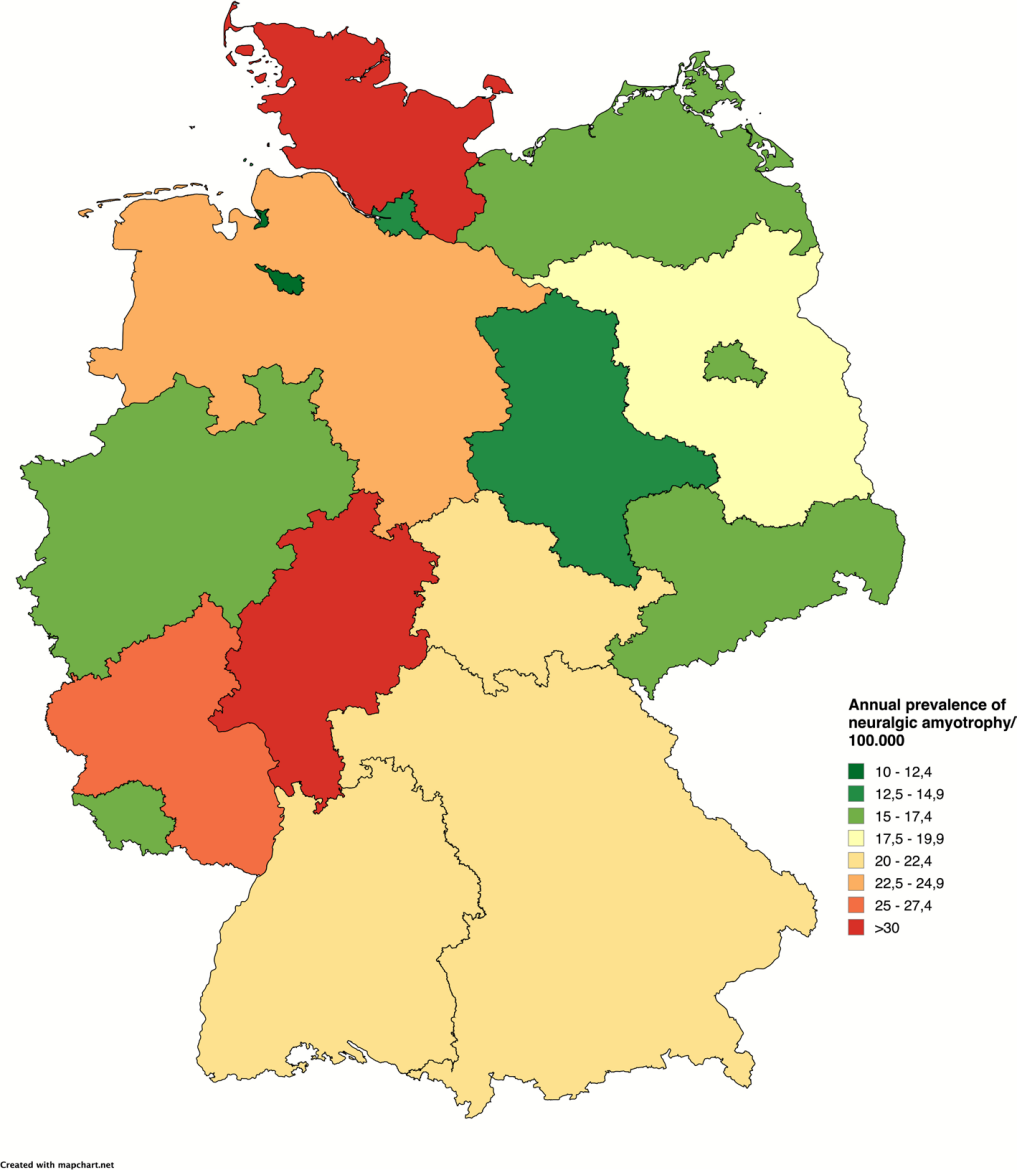
Geographical distribution of NA prevalence in Germany. A lower prevalence is observed in the eastern federal states. Additionally, a slight north–south divide is seen in the western federal states.

The prevalence of NA in the overall population averaged 20.8/100,000 from 2013 to 2022. As shown for the incidence of NA, we also report a significant decline from 21.2 in 2013 to 19.7/100,000 in 2022 (OR per year 0.991; 95% CI [0.988; 0.994]; *p* < 0.001). The highest prevalence was seen in the 50–59 y age group at 33.4/100,000, and the lowest in the ≤ 19 y age group at 2.1/100,000 (Figure 3). A ratio of 1:1.2 men to women was observed in all age groups. The distribution of NA prevalence within Germany is heterogeneous (Figure 4). While the overall rate is lower in the eastern federal states (17.4 vs. 24.3/100,000), a distinct north–south divide emerges in the other federal states, with the highest prevalence rates observed in Hessen (32.5/100,000) and Schleswig‐Holstein (30.1/100,000), whereas it is 21.8 in Bavaria and 16.4/100,000 in North Rhine‐Westphalia.

The prevalence of simultaneously coded diaphragmatic paresis (indicative of phrenic nerve involvement) among insured persons with a coded diagnosis of neuralgic amyotrophy was 0.45% on average (95% CI [0.30; 0.68]), with an increase of 88% in the period studied (Table 1).

## 4 Discussion

Our retrospective analysis revealed an average incidence rate of NA of 10.3 per 100,000 people. This rate is 90% lower than the rate determined prospectively [6]. This discrepancy may be partly due to some NA patients being billed under different ICD codes. Since physicians who bill are required to use the most specific codes possible, the discrepancy is likely primarily due to an incorrect diagnosis in a large proportion of NA cases. Consistent with this, the aforementioned study by van Alfen [6] also found a 97% difference between the previously reported and prospectively determined incidences after general practitioners received training on the clinical symptoms of NA.

Given that timely administration of corticosteroids and surgical intervention for nerve or fascicular torsions are now recognized as standard therapeutic approaches, the potential underdiagnosis of NA highlights a critical gap in early disease recognition and treatment [10, 11, 12]. These findings underscore the need for improved diagnostic strategies and greater awareness to enhance patient outcomes. In this context the prevalence of diaphragmatic paresis in the population studied (0.45%) is substantially lower than the 7.6% reported in the literature [13, 14]. This discrepancy suggests that, outside of controlled studies, over 90% of cases of phrenic nerve involvement in the context of NA may remain undetected.

The heterogeneous distribution of NA prevalence within Germany can be partially explained by differences in coding strategies between the western and eastern federal states. With respect to the pathophysiological concept of NA, however, it is likely that the different incidences of infectious diseases also play a role as possible triggers of the autoimmune process. For example, the incidence of adenovirus infections in the state of Schleswig‐Holstein (0.42/100,000) is higher than that in the states of Bavaria (0.32/100,000) or North Rhine‐Westphalia (0.01/100,000) [15]. A further indication of the decisive role of infectious diseases as triggers of the pathophysiological processes that lead to NA is the significantly higher incidence of NA in the first quarter of the year, in which more respiratory infections occur than in quarters 2–4. Infections of the upper respiratory tract in particular occur more frequently in the winter months and thus follow the seasonal incidence of NA [16]. Similar epidemiological constellations can be found for Guillain‐Barré syndrome (GBS), an immune neuropathy with a pathophysiology that is in part comparable to that of NA [17].

The cause of the steady decline in the incidence and prevalence of NA remains unclear. This is particularly noteworthy given the epidemiology of other autoimmune diseases, which predominantly show increasing incidence and prevalence [18, 19]. These findings suggest that other methodological factors, such as a change in coding guidelines during the study period, may play a role in the case of NA. However, a query of the Federal Office of Public Health's SEG4 database did not reveal any changes to the coding recommendations [20]. In this context, it is also interesting to note that the COVID‐19 pandemic beginning in 2020 and the associated vaccination strategy of the government of the Federal Republic of Germany did not result in an increase in the incidence of NA. This finding stands in contrast to a recent study, which emphasized the relevance of both infection and vaccination with the virus as important immunological triggers of NA [3]. However, measures such as the requirement of masks and repeated lockdowns resulted in a decline in other infectious diseases [15], which may have at least partially offset the effects of COVID‐19. Importantly, the COVID‐19 pandemic has led to a measurably lower frequency of and delays in medical care, which is likely to have influenced the number of coded NA cases [21].

Of the epidemiological studies on NA conducted thus far, approximately two‐thirds of the affected individuals were male, with an average age ranging from 40 to 50 years old [1, 22]. In contrast, our analysis revealed female predominance in all age groups, with a median age of 50 to 59 years for the total collective. This can be primarily explained by the fact that a significant proportion of NA diagnoses are false positives, or type 1 errors. For instance, epidemiological data on adhesive capsulitis of the shoulder joint—an important differential diagnosis of NA—indicate that this condition is significantly more prevalent in women than in men and occurs at an older age overall than NA [23, 24]. However, it should be noted that the prevalence data are based on all insured persons coded with an NA in the corresponding year. It is possible that the diagnosis was permanently coded even after the disease and its consequences subsided. This would primarily affect the prevalence rates in older age groups, which would then be incorrectly increased.

This study has several limitations: Because the International Classification of Diseases, Tenth Edition (ICD‐10) coding system does not contain diagnostic criteria for NA, it is possible that both type I and type II coding errors occurred. Older insured persons are particularly likely to be misdiagnosed, whereby NA is incorrectly classified as conditions with similar clinical symptoms Examples include cervical radiculopathy, rotator cuff tears, and adhesive capsulitis of the shoulder joint. Furthermore, it should be noted that AOK insured persons do not accurately represent the German population in some respects. For instance, 6.8% of AOK insured persons received unemployment benefits during the period under review, compared to 4.1% of all persons with statutory health insurance [25]. Therefore, it is likely that the socioeconomic status of this group is lower than that of the population as a whole.

## 5 Conclusions

The incidence of neuralgic amyotrophy in this analysis of data from a large health insurer was approximately 90% lower than that in prospective surveys. It is likely that the majority of cases are still not correctly diagnosed.

Involvement of the phrenic nerve in patients with NA also appears to be underrecognized.

The current pathophysiological model of NA with an immunological trigger is supported by our data.

In order to better characterize and treat this common disease in the future, it is necessary to establish and validate international diagnostic criteria and conduct further prospective studies based on these criteria. However, raising awareness among physicians is equally important so that more patients can receive early and effective treatment.

## Author Contributions

**Johannes Fabian Holle:** concept development (lead), writing of the original draft (lead), visualization (lead), revision and editing (equal). **Andreas Leha:** statistical data analysis (lead), revision and editing (equal). **Carolin Polte:** statistical data analysis (supporting), revision and editing (equal). **Volker Limmroth:** revision and editing (equal). **Wolfram Windisch:** revision and editing (equal). **Maximilian Zimmermann:** concept development (supporting), writing of the original draft (supporting), revision and editing (equal).

## Ethics Statement

We confirm that we have read the Journal's position on issues involved in ethical publication and affirm that this report is consistent with those guidelines.

## Conflicts of Interest

The authors declare no conflicts of interest.

## Supporting information


**Data S1:** Supporting Information

## Data Availability

The data that supports the findings of this study is available in the Supporting Information of this article.
