# Supplementary material for: Epidemiology of Neuralgic Amyotrophy—A Retrospective Analysis of Data From a Large German Health Insurance Company
Source: Muscle Nerve. 2025 Nov 14;73(1):56–62. doi: 10.1002/mus.70059 (PMC12690013; doi:10.1002/mus.70059)
Supplement: Supplementary file 1 — Data S1: Supporting Information [file MUS-73-56-s001.html]

Diabetes in Patients With and Without Neuralgic Amyotrophy


| **Wissenschaftliche Serviceeinheit** **Medizinische Biometrie und Statistische Bioinformatik** Leitung: Dr. Andreas Leha |


  |
| Dr. med. Johannes Holle  Klinik für Neurologie  Lehrstuhl Pneumologie der Privaten Universität Witten/Herdecke  Kliniken der Stadt Köln gGmbH  Ostmerheimer Str. 200    51109 Köln | Institut für Medizinische Statistik  Direktor: Prof. Dr. Tim Friede |
| **Ihr Ansprechpartner**  Dr. Andreas Leha |
| 37099 Göttingen  **Briefpost**  Humboldtallee 32, 37073 Göttingen  **Adresse**  0551 / 39-64829  **Telefon**  0551 / 39-65605  **Fax**  andreas.leha@med.uni-goettingen.de  **E-Mail** |
|  | MBSB-2024-0032 **Auftragsnummer**  14. März 2025 **Datum** |

# Diabetes in Patients With and Without Neuralgic Amyotrophy

667e631f966aeba7645f7da7d57ce213b8aeaeb4

---

> PLEASE NOTE: This report was authored by the Scientific Core Facility for Medical Biometry and Statistical Bioinformatics (MBSB) of the University Medical Center Göttingen (UMG). In accordance with the Vancouver Recommendations (http://www.icmje.org/recommendations/) and the user agreement of the MBSB, the authors of this report must be acknowledged as co-authors in any publication (e.g. paper, presentation, poster) using any results of this report. If used in a thesis, the contributions of the authors and MBSB must be clearly acknowledged. Any use of the results as well as citation or the naming of MBSB or persons of the MBSB for other purposes (e.g. grant or ethics board application) requires the explicit approval of the contact person. Upon completion, a copy of any work based on this report must be submitted to the contact person at MBSB. MBSB reserves the right to publish corrections regarding results in this report and their interpretation without the consent of the coauthors.

---

# 1 Introduction

The aim of this study is to assess prevalences of diabetes (ICD-Codes E10 and E11) in patients with neuralgic amyotrophy (NA) and in all patients.

# 2 Statistical Methods

Proportions of patients with NA as well as with diabetes were calclulated and presented with 95% confidence intervals among all patients and in subgroups (e.g. among patients with NA) and visualised via barplots. Proportions are scaled to give numbers per 100.000 where indicated.

Numbers of first diagnoses of NA were compared between the first and the remaining yearly quarters by means of a Poisson regression model including year and yearly quarter as predictors. Resulting estimates are presented with 95% confidence interval and p values testing the null hypothesis of no association. Normalization to total numbers of insured patients was not possible as these data were not available. As the total number of patients is not to be expected to fluctuate with quarter, the comparisons of quarters is deemed to be valid nonetheless.

Proportions were tested for trend over time using logistic regression models. Resulting regression coefficients are given as odds ratios with 95% confidence interval and p value testing the null hypothesis of no association. Model predictions with 95% CIs were visualised als line plot. Additionally, χ² test for trend in proportions were carried out as sensitivity analysis.

Proportions of patients with diabetes in each age class were also compared between patients with NA and without NA by means of contrast tests on a logistic regression model using age group and NA as predictors. Results are presented as odds ratios with 95% confidence interval and p value testing the null hypothesis of no difference. For the age group 40-49 only data from years 2016,2017,2018,2020, and 2021 are considered, as the other years had incomplete data.

Proportions of patients with diabetes across all years and age groups were compared using Fisher’s exact test. For this totals were calculated for all age groups 50-59, 60-69, 70-79, >=80 and for age group 40-49 in years 2016,2017,2018,2020,2021 as the data was incomplete for the other years/age groups.

The significance level was set to alpha = 5% for all statistical tests. All analyses were performed with the statistic software R (version 4.4.0; R Core Team 2024) using the R-package `emmeans` (version 1.10.7; Lenth 2025) for the contrast tests.

# 3 Data

Summary data was available for the years 2013-2022 in age groups <=19, 20-29, …, >=80.

# 4 Results

## 4.1 Neuralgic Amyotrophy Over Time

### 4.1.1 First Diagnosis

**Figure 1:** Number of patients with first diagnosis of neuralgic amyotrophy by year and quarter

**Figure 2:** Number of patients with first diagnosis of neuralgic amyotrophy by quarter across all years

**Table 1:** Model coefficients with 95% confidence interval from a Poisson regression model for the number of first NA diagnoses using year and quarter as predictors. Interpretation example: In Q2 there are expected to be 14% (95%-CI [11%-17%]) fewer new NA diagnoses compared to Q1. NOTE: not corrected for number of total cases! Maybe not too harmful for comparison of quarters, but especially for the year comparison!


| term | estimate | ci | p.value |
| --- | --- | --- | --- |
| (Intercept) | 858 | [833; 883] | *< 0.001* |
| Jahr | 0.9615 | [0.96; 0.97] | *< 0.001* |
| Quartal2 | 0.8607 | [0.83; 0.89] | *< 0.001* |
| Quartal3 | 0.8842 | [0.85; 0.91] | *< 0.001* |
| Quartal4 | 0.8758 | [0.85; 0.91] | *< 0.001* |

**Table 2:** Patients per 100.000 with newly diagnosed neuralgic amyotrophy by year


| Jahr | Anzahl\_Versicherte | Anzahl\_Versicherte\_newNA | proportion\_newNA | proportion\_newNA\_ci |
| --- | --- | --- | --- | --- |
| 2013 | 24114347 | 3087 | 12.8 | [12.4; 13.3] |
| 2014 | 24158146 | 2978 | 12.3 | [11.9; 12.8] |
| 2015 | 24302580 | 2877 | 11.8 | [11.4; 12.3] |
| 2016 | 25014139 | 2735 | 10.9 | [10.5; 11.4] |
| 2017 | 25735948 | 2686 | 10.4 | [10; 10.8] |
| 2018 | 26264475 | 2500 | 9.5 | [9.2; 9.9] |
| 2019 | 26477052 | 2577 | 9.7 | [9.4; 10.1] |
| 2020 | 26770605 | 2375 | 8.9 | [8.5; 9.2] |
| 2021 | 26762835 | 2308 | 8.6 | [8.3; 9] |
| 2022 | 26899648 | 2080 | 7.7 | [7.4; 8.1] |

**Table 3:** Patients per 100.000 with newly diagnosed neuralgic amyotrophy – mean over years


| Anzahl\_Versicherte | proportion\_newNA | proportion\_newNA\_ci |
| --- | --- | --- |
| 25649978 | 10.3 | [9.9; 10.7] |

**Table 4:** Patients per 100.000 with newly diagnosed neuralgic amyotrophy by quater. NOTE: quaterly data for the total numbers of patients is not available; the available yearly numbers are here assumed to be fixed across the quarters of the corresponding year.


| Jahr | Quartal | Anzahl\_Versicherte | Anzahl\_Versicherte\_newNA | proportion\_newNA | proportion\_newNA\_ci |
| --- | --- | --- | --- | --- | --- |
| 2013 | 1 | 24114347 | 812 | 13.5 | [13; 13.9] |
| 2013 | 2 | 24114347 | 760 | 12.6 | [12.2; 13.1] |
| 2013 | 3 | 24114347 | 752 | 12.5 | [12; 12.9] |
| 2013 | 4 | 24114347 | 763 | 12.7 | [12.2; 13.1] |
| 2014 | 1 | 24158146 | 878 | 14.5 | [14.1; 15] |
| 2014 | 2 | 24158146 | 658 | 10.9 | [10.5; 11.3] |
| 2014 | 3 | 24158146 | 778 | 12.9 | [12.4; 13.3] |
| 2014 | 4 | 24158146 | 664 | 11 | [10.6; 11.4] |
| 2015 | 1 | 24302580 | 759 | 12.5 | [12.1; 12.9] |
| 2015 | 2 | 24302580 | 653 | 10.7 | [10.3; 11.2] |
| 2015 | 3 | 24302580 | 735 | 12.1 | [11.7; 12.5] |
| 2015 | 4 | 24302580 | 730 | 12 | [11.6; 12.5] |
| 2016 | 1 | 25014139 | 734 | 11.7 | [11.3; 12.2] |
| 2016 | 2 | 25014139 | 710 | 11.4 | [10.9; 11.8] |
| 2016 | 3 | 25014139 | 639 | 10.2 | [9.8; 10.6] |
| 2016 | 4 | 25014139 | 652 | 10.4 | [10; 10.8] |
| 2017 | 1 | 25735948 | 783 | 12.2 | [11.7; 12.6] |
| 2017 | 2 | 25735948 | 662 | 10.3 | [9.9; 10.7] |
| 2017 | 3 | 25735948 | 602 | 9.4 | [9; 9.7] |
| 2017 | 4 | 25735948 | 639 | 9.9 | [9.6; 10.3] |
| 2018 | 1 | 26264475 | 674 | 10.3 | [9.9; 10.7] |
| 2018 | 2 | 26264475 | 591 | 9 | [8.6; 9.4] |
| 2018 | 3 | 26264475 | 605 | 9.2 | [8.9; 9.6] |
| 2018 | 4 | 26264475 | 630 | 9.6 | [9.2; 10] |
| 2019 | 1 | 26477052 | 711 | 10.7 | [10.4; 11.1] |
| 2019 | 2 | 26477052 | 593 | 9 | [8.6; 9.3] |
| 2019 | 3 | 26477052 | 629 | 9.5 | [9.1; 9.9] |
| 2019 | 4 | 26477052 | 644 | 9.7 | [9.4; 10.1] |
| 2020 | 1 | 26770605 | 640 | 9.6 | [9.2; 9.9] |
| 2020 | 2 | 26770605 | 553 | 8.3 | [7.9; 8.6] |
| 2020 | 3 | 26770605 | 612 | 9.1 | [8.8; 9.5] |
| 2020 | 4 | 26770605 | 570 | 8.5 | [8.2; 8.9] |
| 2021 | 1 | 26762835 | 640 | 9.6 | [9.2; 9.9] |
| 2021 | 2 | 26762835 | 546 | 8.2 | [7.8; 8.5] |
| 2021 | 3 | 26762835 | 567 | 8.5 | [8.1; 8.8] |
| 2021 | 4 | 26762835 | 555 | 8.3 | [8; 8.6] |
| 2022 | 1 | 26899648 | 606 | 9 | [8.7; 9.4] |
| 2022 | 2 | 26899648 | 503 | 7.5 | [7.2; 7.8] |
| 2022 | 3 | 26899648 | 480 | 7.1 | [6.8; 7.5] |
| 2022 | 4 | 26899648 | 491 | 7.3 | [7; 7.6] |

**Table 5:** Patients per 100.000 with newly diagnosed neuralgic amyotrophy by quater averaged across years. NOTE: quaterly data for the total numbers of patients is not available; the available yearly numbers are here assumed to be fixed across the quarters of the corresponding year.


| Quartal | Anzahl\_Versicherte | Anzahl\_Versicherte\_newNA | proportion\_newNA | proportion\_newNA\_ci |
| --- | --- | --- | --- | --- |
| 1 | 25649978 | 724 | 11.3 | [10.9; 11.7] |
| 2 | 25649978 | 623 | 9.7 | [9.3; 10.1] |
| 3 | 25649978 | 640 | 10 | [9.6; 10.4] |
| 4 | 25649978 | 634 | 9.9 | [9.5; 10.3] |

**Table 6:** Model coefficients with 95% confidence intervals from a logistic regression model


| term | OR | ci | p.value |
| --- | --- | --- | --- |
| (Intercept) | 0.0001295 | [0.000127; 0.000132] | *< 0.001* |
| Jahr | 0.9476 | [0.944; 0.952] | *< 0.001* |

**Table 7:** Results from a trend test for proportions


| statistic | df | p.value | method |
| --- | --- | --- | --- |
| 622 | 1 | *< 0.001* | Chi-squared Test for Trend in Proportions |

**Figure 3:** Proportion of patients with newly diagnosed neuralgic amyotrophy per 100.000 by year shown with 95% confidence intervals

### 4.1.2 Prevalence

**Table 8:** patients per 100.000 with neuralgic amyotrophy by year and age


| Jahr | Altersgruppe | Anzahl\_Versicherte | Anzahl\_Versicherte\_G54 | proportion\_G54 | proportion\_G54\_ci |
| --- | --- | --- | --- | --- | --- |
| 2013 | <=19 | 4238790 | 102 | 2.4 | [2; 2.9] |
| 2013 | 20-29 | 2996851 | 374 | 12.5 | [11.3; 13.8] |
| 2013 | 30-39 | 2601042 | 517 | 19.9 | [18.2; 21.7] |
| 2013 | 40-49 | 3280323 | 947 | 28.9 | [27.1; 30.8] |
| 2013 | 50-59 | 3479395 | 1160 | 33.3 | [31.5; 35.3] |
| 2013 | 60-69 | 2619472 | 802 | 30.6 | [28.6; 32.8] |
| 2013 | 70-79 | 2950342 | 802 | 27.2 | [25.3; 29.1] |
| 2013 | >=80 | 1948132 | 415 | 21.3 | [19.3; 23.5] |
| 2014 | <=19 | 4233599 | 114 | 2.7 | [2.2; 3.2] |
| 2014 | 20-29 | 3024908 | 361 | 11.9 | [10.7; 13.2] |
| 2014 | 30-39 | 2690645 | 532 | 19.8 | [18.1; 21.5] |
| 2014 | 40-49 | 3177148 | 926 | 29.1 | [27.3; 31.1] |
| 2014 | 50-59 | 3550080 | 1153 | 32.5 | [30.6; 34.4] |
| 2014 | 60-69 | 2651330 | 852 | 32.1 | [30; 34.4] |
| 2014 | 70-79 | 2830921 | 785 | 27.7 | [25.8; 29.8] |
| 2014 | >=80 | 2e+06 | 447 | 22.4 | [20.4; 24.6] |
| 2015 | <=19 | 4274640 | 119 | 2.8 | [2.3; 3.3] |
| 2015 | 20-29 | 3049059 | 355 | 11.6 | [10.5; 12.9] |
| 2015 | 30-39 | 2812026 | 515 | 18.3 | [16.8; 20] |
| 2015 | 40-49 | 3097736 | 822 | 26.5 | [24.8; 28.4] |
| 2015 | 50-59 | 3619640 | 1238 | 34.2 | [32.3; 36.2] |
| 2015 | 60-69 | 2750557 | 918 | 33.4 | [31.3; 35.6] |
| 2015 | 70-79 | 2655048 | 765 | 28.8 | [26.8; 30.9] |
| 2015 | >=80 | 2043874 | 518 | 25.3 | [23.2; 27.6] |
| 2016 | <=19 | 4460627 | 99 | 2.2 | [1.8; 2.7] |
| 2016 | 20-29 | 3174518 | 394 | 12.4 | [11.2; 13.7] |
| 2016 | 30-39 | 3033532 | 563 | 18.6 | [17.1; 20.2] |
| 2016 | 40-49 | 3098764 | 841 | 27.1 | [25.4; 29.1] |
| 2016 | 50-59 | 3744118 | 1277 | 34.1 | [32.3; 36] |
| 2016 | 60-69 | 2877199 | 939 | 32.6 | [30.6; 34.8] |
| 2016 | 70-79 | 2529988 | 750 | 29.6 | [27.6; 31.9] |
| 2016 | >=80 | 2095393 | 494 | 23.6 | [21.6; 25.8] |
| 2017 | <=19 | 4713691 | 100 | 2.1 | [1.7; 2.6] |
| 2017 | 20-29 | 3307627 | 405 | 12.2 | [11.1; 13.5] |
| 2017 | 30-39 | 3265393 | 566 | 17.3 | [15.9; 18.8] |
| 2017 | 40-49 | 3108670 | 820 | 26.4 | [24.6; 28.3] |
| 2017 | 50-59 | 3827844 | 1316 | 34.4 | [32.6; 36.3] |
| 2017 | 60-69 | 2960468 | 985 | 33.3 | [31.2; 35.4] |
| 2017 | 70-79 | 2421742 | 674 | 27.8 | [25.8; 30] |
| 2017 | >=80 | 2130513 | 520 | 24.4 | [22.4; 26.6] |
| 2018 | <=19 | 4896014 | 98 | 2 | [1.6; 2.5] |
| 2018 | 20-29 | 3348365 | 365 | 10.9 | [9.8; 12.1] |
| 2018 | 30-39 | 3463044 | 545 | 15.7 | [14.5; 17.1] |
| 2018 | 40-49 | 3118502 | 806 | 25.8 | [24.1; 27.7] |
| 2018 | 50-59 | 3903857 | 1274 | 32.6 | [30.9; 34.5] |
| 2018 | 60-69 | 3041641 | 1030 | 33.9 | [31.8; 36] |
| 2018 | 70-79 | 2320944 | 637 | 27.4 | [25.4; 29.7] |
| 2018 | >=80 | 2172108 | 560 | 25.8 | [23.7; 28] |
| 2019 | <=19 | 5020305 | 102 | 2 | [1.7; 2.5] |
| 2019 | 20-29 | 3293842 | 361 | 11 | [9.9; 12.2] |
| 2019 | 30-39 | 3569072 | 647 | 18.1 | [16.8; 19.6] |
| 2019 | 40-49 | 3093089 | 773 | 25 | [23.3; 26.8] |
| 2019 | 50-59 | 3920302 | 1307 | 33.3 | [31.6; 35.2] |
| 2019 | 60-69 | 3115215 | 1076 | 34.5 | [32.5; 36.7] |
| 2019 | 70-79 | 2239206 | 622 | 27.8 | [25.7; 30.1] |
| 2019 | >=80 | 2226021 | 563 | 25.3 | [23.3; 27.5] |
| 2020 | <=19 | 5132309 | 83 | 1.6 | [1.3; 2] |
| 2020 | 20-29 | 3269446 | 336 | 10.3 | [9.2; 11.5] |
| 2020 | 30-39 | 3692445 | 630 | 17.1 | [15.8; 18.5] |
| 2020 | 40-49 | 3123483 | 779 | 24.9 | [23.2; 26.8] |
| 2020 | 50-59 | 3915855 | 1289 | 32.9 | [31.2; 34.8] |
| 2020 | 60-69 | 3191659 | 1083 | 33.9 | [32; 36] |
| 2020 | 70-79 | 2179916 | 606 | 27.8 | [25.7; 30.1] |
| 2020 | >=80 | 2265492 | 582 | 25.7 | [23.7; 27.9] |
| 2021 | <=19 | 5178653 | 82 | 1.6 | [1.3; 2] |
| 2021 | 20-29 | 3197663 | 322 | 10.1 | [9; 11.2] |
| 2021 | 30-39 | 3729849 | 628 | 16.8 | [15.6; 18.2] |
| 2021 | 40-49 | 3129605 | 789 | 25.2 | [23.5; 27] |
| 2021 | 50-59 | 3864761 | 1295 | 33.5 | [31.7; 35.4] |
| 2021 | 60-69 | 3264254 | 1115 | 34.2 | [32.2; 36.2] |
| 2021 | 70-79 | 2143264 | 642 | 30 | [27.7; 32.4] |
| 2021 | >=80 | 2254786 | 587 | 26 | [24; 28.2] |
| 2022 | <=19 | 5271965 | 84 | 1.6 | [1.3; 2] |
| 2022 | 20-29 | 3155682 | 288 | 9.1 | [8.1; 10.3] |
| 2022 | 30-39 | 3783600 | 581 | 15.4 | [14.1; 16.7] |
| 2022 | 40-49 | 3199011 | 742 | 23.2 | [21.6; 24.9] |
| 2022 | 50-59 | 3793614 | 1243 | 32.8 | [31; 34.7] |
| 2022 | 60-69 | 3339987 | 1139 | 34.1 | [32.2; 36.2] |
| 2022 | 70-79 | 2163197 | 647 | 29.9 | [27.7; 32.3] |
| 2022 | >=80 | 2192592 | 584 | 26.6 | [24.5; 28.9] |

**Figure 4:** Proportion of patients with neuralgic amyotrophy per 100.000 by year and age (black lines) shown with 95% confidence intervals (error bars). The blue line show the model predictions from a logistic regression model neuralgic amyotrophy with confidence intervals around the model predictions displayed as shaded area.

**Table 9:** Model coefficients with 95% confidence intervals from a logistic regression model


| term | OR | ci | p.value |
| --- | --- | --- | --- |
| (Intercept) | 0.0002163 | [0.0002129; 0.0002198] | *< 0.001* |
| Jahr | 0.9909 | [0.9880; 0.9939] | *< 0.001* |

**Table 10:** Results from a trend test for proportions


| statistic | df | p.value | method |
| --- | --- | --- | --- |
| 36.07 | 1 | *< 0.001* | Chi-squared Test for Trend in Proportions |

**Table 11:** patients per 100.000 with neuralgic amyotrophy by year


| Jahr | Anzahl\_Versicherte | Anzahl\_Versicherte\_G54 | proportion\_G54 | proportion\_G54\_ci |
| --- | --- | --- | --- | --- |
| 2013 | 24114347 | 5119 | 21.2 | [20.7; 21.8] |
| 2014 | 24158146 | 5170 | 21.4 | [20.8; 22] |
| 2015 | 24302580 | 5250 | 21.6 | [21; 22.2] |
| 2016 | 25014139 | 5357 | 21.4 | [20.8; 22] |
| 2017 | 25735948 | 5386 | 20.9 | [20.4; 21.5] |
| 2018 | 26264475 | 5315 | 20.2 | [19.7; 20.8] |
| 2019 | 26477052 | 5451 | 20.6 | [20; 21.1] |
| 2020 | 26770605 | 5388 | 20.1 | [19.6; 20.7] |
| 2021 | 26762835 | 5460 | 20.4 | [19.9; 21] |
| 2022 | 26899648 | 5308 | 19.7 | [19.2; 20.3] |

**Table 12:** Patients per 100.000 with neuralgic amyotrophy – mean over years


| Anzahl\_Versicherte | proportion\_G54 | proportion\_G54\_ci |
| --- | --- | --- |
| 25649978 | 20.7 | [20.2; 21.3] |

**Figure 5:** Proportion of patients with neuralgic amyotrophy per 100.000 by year shown with 95% confidence intervals

**Table 13:** patients per 100.000 with neuralgic amyotrophy by age – mean across years


| Altersgruppe | Anzahl\_Versicherte | proportion\_G54 | proportion\_G54\_ci |
| --- | --- | --- | --- |
| <=19 | 4742059 | 2.1 | [1.7; 2.6] |
| 20-29 | 3181796 | 11.2 | [10.1; 12.4] |
| 30-39 | 3264065 | 17.7 | [16.3; 19.2] |
| 40-49 | 3142633 | 26.2 | [24.5; 28.1] |
| 50-59 | 3761947 | 33.4 | [31.6; 35.3] |
| 60-69 | 2981178 | 33.3 | [31.3; 35.4] |
| 70-79 | 2443457 | 28.4 | [26.3; 30.6] |
| >=80 | 2132843 | 24.7 | [22.6; 26.9] |

**Figure 6:** Proportion of patients with neuralgic amyotrophy per 100.000 by age shown with 95% confidence intervals

## 4.2 Zwerchfellparese

**Table 14:** Percentage of patients with J98.6 in patients with neuralgic amyotrophy (NA prevalence)


| Jahr | Anzahl\_Versicherte\_NA | J98.6 | percentage\_J98 | 95% CI |
| --- | --- | --- | --- | --- |
| 2013 | 5119 | 14 | 0.3 | [0.2; 0.5] |
| 2014 | 5170 | 16 | 0.3 | [0.2; 0.5] |
| 2015 | 5250 | 19 | 0.4 | [0.2; 0.6] |
| 2016 | 5357 | 26 | 0.5 | [0.3; 0.7] |
| 2017 | 5386 | 24 | 0.4 | [0.3; 0.7] |
| 2018 | 5315 | 29 | 0.5 | [0.4; 0.8] |
| 2019 | 5451 | 27 | 0.5 | [0.3; 0.7] |
| 2020 | 5388 | 26 | 0.5 | [0.3; 0.7] |
| 2021 | 5460 | 29 | 0.5 | [0.4; 0.8] |
| 2022 | 5308 | 31 | 0.6 | [0.4; 0.8] |

**Table 15:** Percentage of patients with J98.6 in patients with neuralgic amyotrophy (NA prevalence) – mean over years


| Anzahl\_Versicherte\_NA | percentage\_J98 | 95% CI |
| --- | --- | --- |
| 5320 | 0.4511 | [0.3; 0.68] |

## 4.3 Diabetes

**Table 16:** Proportion of E10/E11 in patients with neuralgic amyotrophy by year, sex, and age


| Jahr | Geschlecht | Altersgruppe | Anzahl\_Versicherte\_G54 | Anzahl\_Versicherte\_G54\_E10E11 | G54\_proportion\_E1011 | G54\_proportion\_E1011\_ci |
| --- | --- | --- | --- | --- | --- | --- |
| 2013 | m | <=19 | 43 | <30 | <0.7 | [0; <0.82] |
| 2013 | m | 20-29 | 174 | <30 | <0.17 | [0; <0.24] |
| 2013 | m | 30-39 | 229 | <30 | <0.13 | [0; <0.18] |
| 2013 | m | 40-49 | 429 | 39 | 0.09 | [0.07; 0.12] |
| 2013 | m | 50-59 | 538 | 98 | 0.18 | [0.15; 0.22] |
| 2013 | m | 60-69 | 380 | 115 | 0.3 | [0.26; 0.35] |
| 2013 | m | 70-79 | 340 | 129 | 0.38 | [0.33; 0.43] |
| 2013 | m | >=80 | 120 | 47 | 0.39 | [0.31; 0.49] |
| 2013 | w | <=19 | 59 | 0 | 0 | [0; 0.08] |
| 2013 | w | 20-29 | 200 | <30 | <0.15 | [0; <0.21] |
| 2013 | w | 30-39 | 288 | <30 | <0.1 | [0; <0.15] |
| 2013 | w | 40-49 | 518 | <30 | <0.06 | [0; <0.08] |
| 2013 | w | 50-59 | 622 | 78 | 0.13 | [0.1; 0.15] |
| 2013 | w | 60-69 | 422 | 119 | 0.28 | [0.24; 0.33] |
| 2013 | w | 70-79 | 462 | 171 | 0.37 | [0.33; 0.42] |
| 2013 | w | >=80 | 295 | 104 | 0.35 | [0.3; 0.41] |
| 2014 | m | <=19 | 44 | 0 | 0 | [0; 0.1] |
| 2014 | m | 20-29 | 154 | <30 | <0.19 | [0; <0.27] |
| 2014 | m | 30-39 | 236 | <30 | <0.13 | [0; <0.18] |
| 2014 | m | 40-49 | 421 | 37 | 0.09 | [0.06; 0.12] |
| 2014 | m | 50-59 | 539 | 96 | 0.18 | [0.15; 0.21] |
| 2014 | m | 60-69 | 417 | 140 | 0.34 | [0.29; 0.38] |
| 2014 | m | 70-79 | 350 | 144 | 0.41 | [0.36; 0.47] |
| 2014 | m | >=80 | 140 | 61 | 0.44 | [0.35; 0.52] |
| 2014 | w | <=19 | 70 | 0 | 0 | [0; 0.06] |
| 2014 | w | 20-29 | 207 | <30 | <0.14 | [0; <0.2] |
| 2014 | w | 30-39 | 296 | <30 | <0.1 | [0; <0.14] |
| 2014 | w | 40-49 | 505 | <30 | <0.06 | [0; <0.08] |
| 2014 | w | 50-59 | 614 | 79 | 0.13 | [0.1; 0.16] |
| 2014 | w | 60-69 | 435 | 121 | 0.28 | [0.24; 0.32] |
| 2014 | w | 70-79 | 435 | 158 | 0.36 | [0.32; 0.41] |
| 2014 | w | >=80 | 307 | 112 | 0.36 | [0.31; 0.42] |
| 2015 | m | <=19 | 49 | 0 | 0 | [0; 0.09] |
| 2015 | m | 20-29 | 160 | <30 | <0.19 | [0; <0.26] |
| 2015 | m | 30-39 | 226 | <30 | <0.13 | [0; <0.19] |
| 2015 | m | 40-49 | 372 | <30 | <0.08 | [0; <0.11] |
| 2015 | m | 50-59 | 569 | 95 | 0.17 | [0.14; 0.2] |
| 2015 | m | 60-69 | 450 | 145 | 0.32 | [0.28; 0.37] |
| 2015 | m | 70-79 | 305 | 124 | 0.41 | [0.35; 0.46] |
| 2015 | m | >=80 | 177 | 77 | 0.44 | [0.36; 0.51] |
| 2015 | w | <=19 | 70 | 0 | 0 | [0; 0.06] |
| 2015 | w | 20-29 | 195 | <30 | <0.15 | [0; <0.21] |
| 2015 | w | 30-39 | 289 | <30 | <0.1 | [0; <0.15] |
| 2015 | w | 40-49 | 450 | 34 | 0.08 | [0.05; 0.1] |
| 2015 | w | 50-59 | 669 | 77 | 0.12 | [0.09; 0.14] |
| 2015 | w | 60-69 | 468 | 117 | 0.25 | [0.21; 0.29] |
| 2015 | w | 70-79 | 460 | 156 | 0.34 | [0.3; 0.38] |
| 2015 | w | >=80 | 341 | 121 | 0.35 | [0.3; 0.41] |
| 2016 | m | <=19 | 42 | 0 | 0 | [0; 0.1] |
| 2016 | m | 20-29 | 187 | <30 | <0.16 | [0; <0.22] |
| 2016 | m | 30-39 | 258 | <30 | <0.12 | [0; <0.16] |
| 2016 | m | 40-49 | 390 | 39 | 0.1 | [0.07; 0.14] |
| 2016 | m | 50-59 | 606 | 106 | 0.17 | [0.15; 0.21] |
| 2016 | m | 60-69 | 461 | 149 | 0.32 | [0.28; 0.37] |
| 2016 | m | 70-79 | 290 | 108 | 0.37 | [0.32; 0.43] |
| 2016 | m | >=80 | 165 | 60 | 0.36 | [0.29; 0.44] |
| 2016 | w | <=19 | 57 | 0 | 0 | [0; 0.08] |
| 2016 | w | 20-29 | 207 | <30 | <0.14 | [0; <0.2] |
| 2016 | w | 30-39 | 305 | <30 | <0.1 | [0; <0.14] |
| 2016 | w | 40-49 | 451 | 38 | 0.08 | [0.06; 0.11] |
| 2016 | w | 50-59 | 671 | 78 | 0.12 | [0.09; 0.14] |
| 2016 | w | 60-69 | 478 | 117 | 0.24 | [0.21; 0.29] |
| 2016 | w | 70-79 | 460 | 170 | 0.37 | [0.33; 0.42] |
| 2016 | w | >=80 | 329 | 124 | 0.38 | [0.32; 0.43] |
| 2017 | m | <=19 | 46 | 0 | 0 | [0; 0.1] |
| 2017 | m | 20-29 | 183 | <30 | <0.16 | [0; <0.23] |
| 2017 | m | 30-39 | 241 | <30 | <0.12 | [0; <0.17] |
| 2017 | m | 40-49 | 377 | 38 | 0.1 | [0.07; 0.14] |
| 2017 | m | 50-59 | 646 | 113 | 0.17 | [0.15; 0.21] |
| 2017 | m | 60-69 | 498 | 153 | 0.31 | [0.27; 0.35] |
| 2017 | m | 70-79 | 278 | 110 | 0.4 | [0.34; 0.46] |
| 2017 | m | >=80 | 183 | 61 | 0.33 | [0.27; 0.41] |
| 2017 | w | <=19 | 54 | 0 | 0 | [0; 0.08] |
| 2017 | w | 20-29 | 222 | <30 | <0.14 | [0; <0.19] |
| 2017 | w | 30-39 | 325 | <30 | <0.09 | [0; <0.13] |
| 2017 | w | 40-49 | 443 | 32 | 0.07 | [0.05; 0.1] |
| 2017 | w | 50-59 | 670 | 77 | 0.11 | [0.09; 0.14] |
| 2017 | w | 60-69 | 487 | 119 | 0.24 | [0.21; 0.29] |
| 2017 | w | 70-79 | 396 | 147 | 0.37 | [0.32; 0.42] |
| 2017 | w | >=80 | 337 | 128 | 0.38 | [0.33; 0.43] |
| 2018 | m | <=19 | 52 | 0 | 0 | [0; 0.09] |
| 2018 | m | 20-29 | 172 | <30 | <0.17 | [0; <0.24] |
| 2018 | m | 30-39 | 248 | <30 | <0.12 | [0; <0.17] |
| 2018 | m | 40-49 | 372 | 41 | 0.11 | [0.08; 0.15] |
| 2018 | m | 50-59 | 610 | 117 | 0.19 | [0.16; 0.23] |
| 2018 | m | 60-69 | 531 | 173 | 0.33 | [0.29; 0.37] |
| 2018 | m | 70-79 | 270 | 108 | 0.4 | [0.34; 0.46] |
| 2018 | m | >=80 | 192 | 69 | 0.36 | [0.29; 0.43] |
| 2018 | w | <=19 | 46 | 0 | 0 | [0; 0.1] |
| 2018 | w | 20-29 | 193 | <30 | <0.16 | [0; <0.22] |
| 2018 | w | 30-39 | 297 | <30 | <0.1 | [0; <0.14] |
| 2018 | w | 40-49 | 434 | 30 | 0.07 | [0.05; 0.1] |
| 2018 | w | 50-59 | 664 | 85 | 0.13 | [0.1; 0.16] |
| 2018 | w | 60-69 | 499 | 120 | 0.24 | [0.2; 0.28] |
| 2018 | w | 70-79 | 367 | 137 | 0.37 | [0.32; 0.43] |
| 2018 | w | >=80 | 368 | 139 | 0.38 | [0.33; 0.43] |
| 2019 | m | <=19 | 52 | <30 | <0.58 | [0; <0.71] |
| 2019 | m | 20-29 | 166 | <30 | <0.18 | [0; <0.25] |
| 2019 | m | 30-39 | 322 | <30 | <0.09 | [0; <0.13] |
| 2019 | m | 40-49 | 362 | 31 | 0.09 | [0.06; 0.12] |
| 2019 | m | 50-59 | 634 | 106 | 0.17 | [0.14; 0.2] |
| 2019 | m | 60-69 | 574 | 157 | 0.27 | [0.24; 0.31] |
| 2019 | m | 70-79 | 276 | 114 | 0.41 | [0.35; 0.47] |
| 2019 | m | >=80 | 192 | 70 | 0.36 | [0.3; 0.44] |
| 2019 | w | <=19 | 50 | <30 | <0.6 | [0; <0.73] |
| 2019 | w | 20-29 | 195 | <30 | <0.15 | [0; <0.21] |
| 2019 | w | 30-39 | 325 | <30 | <0.09 | [0; <0.13] |
| 2019 | w | 40-49 | 411 | <30 | <0.07 | [0; <0.1] |
| 2019 | w | 50-59 | 673 | 86 | 0.13 | [0.1; 0.16] |
| 2019 | w | 60-69 | 502 | 111 | 0.22 | [0.19; 0.26] |
| 2019 | w | 70-79 | 346 | 124 | 0.36 | [0.31; 0.41] |
| 2019 | w | >=80 | 371 | 130 | 0.35 | [0.3; 0.4] |
| 2020 | m | <=19 | 37 | <30 | <0.81 | [0; <0.91] |
| 2020 | m | 20-29 | 175 | <30 | <0.17 | [0; <0.24] |
| 2020 | m | 30-39 | 313 | <30 | <0.1 | [0; <0.14] |
| 2020 | m | 40-49 | 380 | 30 | 0.08 | [0.05; 0.11] |
| 2020 | m | 50-59 | 652 | 115 | 0.18 | [0.15; 0.21] |
| 2020 | m | 60-69 | 555 | 170 | 0.31 | [0.27; 0.35] |
| 2020 | m | 70-79 | 269 | 105 | 0.39 | [0.33; 0.45] |
| 2020 | m | >=80 | 206 | 84 | 0.41 | [0.34; 0.48] |
| 2020 | w | <=19 | 46 | 0 | 0 | [0; 0.1] |
| 2020 | w | 20-29 | 161 | <30 | <0.19 | [0; <0.26] |
| 2020 | w | 30-39 | 317 | <30 | <0.09 | [0; <0.13] |
| 2020 | w | 40-49 | 399 | 32 | 0.08 | [0.06; 0.11] |
| 2020 | w | 50-59 | 637 | 89 | 0.14 | [0.11; 0.17] |
| 2020 | w | 60-69 | 528 | 121 | 0.23 | [0.19; 0.27] |
| 2020 | w | 70-79 | 337 | 118 | 0.35 | [0.3; 0.4] |
| 2020 | w | >=80 | 376 | 126 | 0.34 | [0.29; 0.39] |
| 2021 | m | <=19 | 41 | <30 | <0.73 | [0; <0.85] |
| 2021 | m | 20-29 | 157 | 0 | 0 | [0; 0.03] |
| 2021 | m | 30-39 | 293 | <30 | <0.1 | [0; <0.14] |
| 2021 | m | 40-49 | 382 | 36 | 0.09 | [0.07; 0.13] |
| 2021 | m | 50-59 | 615 | 105 | 0.17 | [0.14; 0.2] |
| 2021 | m | 60-69 | 570 | 182 | 0.32 | [0.28; 0.36] |
| 2021 | m | 70-79 | 304 | 123 | 0.4 | [0.35; 0.46] |
| 2021 | m | >=80 | 213 | 75 | 0.35 | [0.29; 0.42] |
| 2021 | w | <=19 | 41 | 0 | 0 | [0; 0.11] |
| 2021 | w | 20-29 | 165 | <30 | <0.18 | [0; <0.25] |
| 2021 | w | 30-39 | 335 | <30 | <0.09 | [0; <0.13] |
| 2021 | w | 40-49 | 407 | 34 | 0.08 | [0.06; 0.12] |
| 2021 | w | 50-59 | 680 | 77 | 0.11 | [0.09; 0.14] |
| 2021 | w | 60-69 | 545 | 130 | 0.24 | [0.2; 0.28] |
| 2021 | w | 70-79 | 338 | 114 | 0.34 | [0.29; 0.39] |
| 2021 | w | >=80 | 374 | 128 | 0.34 | [0.29; 0.39] |
| 2022 | m | <=19 | 46 | <30 | <0.65 | [0; <0.78] |
| 2022 | m | 20-29 | 140 | <30 | <0.21 | [0; <0.29] |
| 2022 | m | 30-39 | 301 | <30 | <0.1 | [0; <0.14] |
| 2022 | m | 40-49 | 391 | 42 | 0.11 | [0.08; 0.14] |
| 2022 | m | 50-59 | 618 | 109 | 0.18 | [0.15; 0.21] |
| 2022 | m | 60-69 | 577 | 191 | 0.33 | [0.29; 0.37] |
| 2022 | m | 70-79 | 318 | 131 | 0.41 | [0.36; 0.47] |
| 2022 | m | >=80 | 202 | 71 | 0.35 | [0.29; 0.42] |
| 2022 | w | <=19 | 38 | 0 | 0 | [0; 0.11] |
| 2022 | w | 20-29 | 148 | <30 | <0.2 | [0; <0.28] |
| 2022 | w | 30-39 | 280 | <30 | <0.11 | [0; <0.15] |
| 2022 | w | 40-49 | 351 | <30 | <0.09 | [0; <0.12] |
| 2022 | w | 50-59 | 625 | 75 | 0.12 | [0.1; 0.15] |
| 2022 | w | 60-69 | 562 | 136 | 0.24 | [0.21; 0.28] |
| 2022 | w | 70-79 | 329 | 104 | 0.32 | [0.27; 0.37] |
| 2022 | w | >=80 | 382 | 136 | 0.36 | [0.31; 0.41] |

**Figure 7:** Proportion of E10/E11 in patients with neuralgic amyotrophy by year, sex, and age shown with 95% confidence intervals

**Table 17:** Proportion of E10/E11 in patients with neuralgic amyotrophy and in all patients by year and age


| Jahr | Altersgruppe | Anzahl\_Versicherte\_gesamt | Anzahl\_Versicherte\_gesamt\_E10E11 | gesamt\_proportion\_E1011 | gesamt\_proportion\_E1011\_conf\_low | gesamt\_proportion\_E1011\_conf\_high | Anzahl\_Versicherte\_G54 | Anzahl\_Versicherte\_G54\_E10E11 | G54\_proportion\_E1011 | G54\_proportion\_E1011\_conf\_low | G54\_proportion\_E1011\_conf\_high |
| --- | --- | --- | --- | --- | --- | --- | --- | --- | --- | --- | --- |
| 2013 | <=19 | 4238790 | 14623 | 0.00345 | 0.0034 | 0.0035 | 102 | NA | NA | NA | NA |
| 2013 | 20-29 | 2996851 | 26443 | 0.008824 | 0.0087 | 0.0089 | 374 | NA | NA | NA | NA |
| 2013 | 30-39 | 2601042 | 56096 | 0.02157 | 0.0214 | 0.0217 | 517 | NA | NA | NA | NA |
| 2013 | 40-49 | 3280323 | 196236 | 0.05982 | 0.0596 | 0.0601 | 947 | NA | NA | NA | NA |
| 2013 | 50-59 | 3479395 | 478971 | 0.1377 | 0.1373 | 0.138 | 1160 | 176 | 0.1517 | 0.1318 | 0.174 |
| 2013 | 60-69 | 2619472 | 706921 | 0.2699 | 0.2693 | 0.2704 | 802 | 234 | 0.2918 | 0.2608 | 0.3248 |
| 2013 | 70-79 | 2950342 | 1091238 | 0.3699 | 0.3693 | 0.3704 | 802 | 300 | 0.3741 | 0.3406 | 0.4087 |
| 2013 | >=80 | 1948132 | 760154 | 0.3902 | 0.3895 | 0.3909 | 415 | 151 | 0.3639 | 0.3179 | 0.4124 |
| 2014 | <=19 | 4233599 | 14841 | 0.003506 | 0.0034 | 0.0036 | 114 | 0 | 0 | 0 | 0.0406 |
| 2014 | 20-29 | 3024908 | 27192 | 0.008989 | 0.0089 | 0.0091 | 361 | NA | NA | NA | NA |
| 2014 | 30-39 | 2690645 | 57773 | 0.02147 | 0.0213 | 0.0216 | 532 | NA | NA | NA | NA |
| 2014 | 40-49 | 3177148 | 192257 | 0.06051 | 0.0603 | 0.0608 | 926 | NA | NA | NA | NA |
| 2014 | 50-59 | 3550080 | 486213 | 0.137 | 0.1366 | 0.1373 | 1153 | 175 | 0.1518 | 0.1318 | 0.1741 |
| 2014 | 60-69 | 2651330 | 714079 | 0.2693 | 0.2688 | 0.2699 | 852 | 261 | 0.3063 | 0.2757 | 0.3387 |
| 2014 | 70-79 | 2830921 | 1050188 | 0.371 | 0.3704 | 0.3715 | 785 | 302 | 0.3847 | 0.3507 | 0.4199 |
| 2014 | >=80 | 2e+06 | 790716 | 0.3955 | 0.3948 | 0.3961 | 447 | 173 | 0.387 | 0.3419 | 0.4341 |
| 2015 | <=19 | 4274640 | 14995 | 0.003508 | 0.0035 | 0.0036 | 119 | 0 | 0 | 0 | 0.039 |
| 2015 | 20-29 | 3049059 | 27906 | 0.009152 | 0.009 | 0.0093 | 355 | NA | NA | NA | NA |
| 2015 | 30-39 | 2812026 | 60734 | 0.0216 | 0.0214 | 0.0218 | 515 | NA | NA | NA | NA |
| 2015 | 40-49 | 3097736 | 188841 | 0.06096 | 0.0607 | 0.0612 | 822 | NA | NA | NA | NA |
| 2015 | 50-59 | 3619640 | 494308 | 0.1366 | 0.1362 | 0.1369 | 1238 | 172 | 0.1389 | 0.1204 | 0.1597 |
| 2015 | 60-69 | 2750557 | 742373 | 0.2699 | 0.2694 | 0.2704 | 918 | 262 | 0.2854 | 0.2566 | 0.316 |
| 2015 | 70-79 | 2655048 | 992330 | 0.3738 | 0.3732 | 0.3743 | 765 | 280 | 0.366 | 0.332 | 0.4014 |
| 2015 | >=80 | 2043874 | 827258 | 0.4047 | 0.4041 | 0.4054 | 518 | 198 | 0.3822 | 0.3405 | 0.4258 |
| 2016 | <=19 | 4460627 | 15290 | 0.003428 | 0.0034 | 0.0035 | 99 | 0 | 0 | 0 | 0.0465 |
| 2016 | 20-29 | 3174518 | 29052 | 0.009152 | 0.009 | 0.0093 | 394 | NA | NA | NA | NA |
| 2016 | 30-39 | 3033532 | 64876 | 0.02139 | 0.0212 | 0.0215 | 563 | NA | NA | NA | NA |
| 2016 | 40-49 | 3098764 | 189050 | 0.06101 | 0.0607 | 0.0613 | 841 | 77 | 0.09156 | 0.0733 | 0.1136 |
| 2016 | 50-59 | 3744118 | 508694 | 0.1359 | 0.1355 | 0.1362 | 1277 | 184 | 0.1441 | 0.1255 | 0.1648 |
| 2016 | 60-69 | 2877199 | 774296 | 0.2691 | 0.2686 | 0.2696 | 939 | 266 | 0.2833 | 0.2549 | 0.3135 |
| 2016 | 70-79 | 2529988 | 951297 | 0.376 | 0.3754 | 0.3766 | 750 | 278 | 0.3707 | 0.3362 | 0.4065 |
| 2016 | >=80 | 2095393 | 856424 | 0.4087 | 0.4081 | 0.4094 | 494 | 184 | 0.3725 | 0.33 | 0.417 |
| 2017 | <=19 | 4713691 | 15510 | 0.00329 | 0.0032 | 0.0033 | 100 | 0 | 0 | 0 | 0.0461 |
| 2017 | 20-29 | 3307627 | 29857 | 0.009027 | 0.0089 | 0.0091 | 405 | NA | NA | NA | NA |
| 2017 | 30-39 | 3265393 | 68618 | 0.02101 | 0.0209 | 0.0212 | 566 | NA | NA | NA | NA |
| 2017 | 40-49 | 3108670 | 189235 | 0.06087 | 0.0606 | 0.0611 | 820 | 70 | 0.08537 | 0.0676 | 0.1071 |
| 2017 | 50-59 | 3827844 | 514835 | 0.1345 | 0.1342 | 0.1348 | 1316 | 190 | 0.1444 | 0.1261 | 0.1648 |
| 2017 | 60-69 | 2960468 | 785945 | 0.2655 | 0.265 | 0.266 | 985 | 272 | 0.2761 | 0.2486 | 0.3054 |
| 2017 | 70-79 | 2421742 | 910556 | 0.376 | 0.3754 | 0.3766 | 674 | 257 | 0.3813 | 0.3447 | 0.4193 |
| 2017 | >=80 | 2130513 | 875975 | 0.4112 | 0.4105 | 0.4118 | 520 | 189 | 0.3635 | 0.3223 | 0.4066 |
| 2018 | <=19 | 4896014 | 15589 | 0.003184 | 0.0031 | 0.0032 | 98 | 0 | 0 | 0 | 0.047 |
| 2018 | 20-29 | 3348365 | 29974 | 0.008952 | 0.0089 | 0.0091 | 365 | NA | NA | NA | NA |
| 2018 | 30-39 | 3463044 | 71677 | 0.0207 | 0.0205 | 0.0208 | 545 | NA | NA | NA | NA |
| 2018 | 40-49 | 3118502 | 186560 | 0.05982 | 0.0596 | 0.0601 | 806 | 71 | 0.08809 | 0.0699 | 0.1103 |
| 2018 | 50-59 | 3903857 | 517575 | 0.1326 | 0.1322 | 0.1329 | 1274 | 202 | 0.1586 | 0.1392 | 0.18 |
| 2018 | 60-69 | 3041641 | 788018 | 0.2591 | 0.2586 | 0.2596 | 1030 | 293 | 0.2845 | 0.2573 | 0.3133 |
| 2018 | 70-79 | 2320944 | 865634 | 0.373 | 0.3723 | 0.3736 | 637 | 245 | 0.3846 | 0.3469 | 0.4238 |
| 2018 | >=80 | 2172108 | 891082 | 0.4102 | 0.4096 | 0.4109 | 560 | 208 | 0.3714 | 0.3315 | 0.4131 |
| 2019 | <=19 | 5020305 | 15990 | 0.003185 | 0.0031 | 0.0032 | 102 | NA | NA | NA | NA |
| 2019 | 20-29 | 3293842 | 30549 | 0.009275 | 0.0092 | 0.0094 | 361 | NA | NA | NA | NA |
| 2019 | 30-39 | 3569072 | 76392 | 0.0214 | 0.0213 | 0.0216 | 647 | NA | NA | NA | NA |
| 2019 | 40-49 | 3093089 | 188362 | 0.0609 | 0.0606 | 0.0612 | 773 | NA | NA | NA | NA |
| 2019 | 50-59 | 3920302 | 525632 | 0.1341 | 0.1337 | 0.1344 | 1307 | 192 | 0.1469 | 0.1284 | 0.1675 |
| 2019 | 60-69 | 3115215 | 797930 | 0.2561 | 0.2557 | 0.2566 | 1076 | 268 | 0.2491 | 0.2237 | 0.2763 |
| 2019 | 70-79 | 2239206 | 837380 | 0.374 | 0.3733 | 0.3746 | 622 | 238 | 0.3826 | 0.3445 | 0.4223 |
| 2019 | >=80 | 2226021 | 909902 | 0.4088 | 0.4081 | 0.4094 | 563 | 200 | 0.3552 | 0.316 | 0.3965 |
| 2020 | <=19 | 5132309 | 16259 | 0.003168 | 0.0031 | 0.0032 | 83 | NA | NA | NA | NA |
| 2020 | 20-29 | 3269446 | 30507 | 0.009331 | 0.0092 | 0.0094 | 336 | NA | NA | NA | NA |
| 2020 | 30-39 | 3692445 | 78000 | 0.02112 | 0.021 | 0.0213 | 630 | NA | NA | NA | NA |
| 2020 | 40-49 | 3123483 | 186361 | 0.05966 | 0.0594 | 0.0599 | 779 | 62 | 0.07959 | 0.062 | 0.1014 |
| 2020 | 50-59 | 3915855 | 517380 | 0.1321 | 0.1318 | 0.1325 | 1289 | 204 | 0.1583 | 0.139 | 0.1796 |
| 2020 | 60-69 | 3191659 | 792561 | 0.2483 | 0.2478 | 0.2488 | 1083 | 291 | 0.2687 | 0.2427 | 0.2964 |
| 2020 | 70-79 | 2179916 | 800744 | 0.3673 | 0.3667 | 0.368 | 606 | 223 | 0.368 | 0.3297 | 0.4079 |
| 2020 | >=80 | 2265492 | 914832 | 0.4038 | 0.4032 | 0.4045 | 582 | 210 | 0.3608 | 0.322 | 0.4015 |
| 2021 | <=19 | 5178653 | 17399 | 0.00336 | 0.0033 | 0.0034 | 82 | NA | NA | NA | NA |
| 2021 | 20-29 | 3197663 | 31706 | 0.009915 | 0.0098 | 0.01 | 322 | NA | NA | NA | NA |
| 2021 | 30-39 | 3729849 | 83218 | 0.02231 | 0.0222 | 0.0225 | 628 | NA | NA | NA | NA |
| 2021 | 40-49 | 3129605 | 190803 | 0.06097 | 0.0607 | 0.0612 | 789 | 70 | 0.08872 | 0.0703 | 0.1113 |
| 2021 | 50-59 | 3864761 | 521736 | 0.135 | 0.1347 | 0.1353 | 1295 | 182 | 0.1405 | 0.1223 | 0.1609 |
| 2021 | 60-69 | 3264254 | 803116 | 0.246 | 0.2456 | 0.2465 | 1115 | 312 | 0.2798 | 0.2538 | 0.3074 |
| 2021 | 70-79 | 2143264 | 782837 | 0.3653 | 0.3646 | 0.3659 | 642 | 237 | 0.3692 | 0.332 | 0.408 |
| 2021 | >=80 | 2254786 | 905040 | 0.4014 | 0.4007 | 0.402 | 587 | 203 | 0.3458 | 0.3076 | 0.3861 |
| 2022 | <=19 | 5271965 | 18543 | 0.003517 | 0.0035 | 0.0036 | 84 | NA | NA | NA | NA |
| 2022 | 20-29 | 3155682 | 32202 | 0.0102 | 0.0101 | 0.0103 | 288 | NA | NA | NA | NA |
| 2022 | 30-39 | 3783600 | 86086 | 0.02275 | 0.0226 | 0.0229 | 581 | NA | NA | NA | NA |
| 2022 | 40-49 | 3199011 | 194051 | 0.06066 | 0.0604 | 0.0609 | 742 | NA | NA | NA | NA |
| 2022 | 50-59 | 3793614 | 516539 | 0.1362 | 0.1358 | 0.1365 | 1243 | 184 | 0.148 | 0.129 | 0.1693 |
| 2022 | 60-69 | 3339987 | 809453 | 0.2424 | 0.2419 | 0.2428 | 1139 | 327 | 0.2871 | 0.2611 | 0.3145 |
| 2022 | 70-79 | 2163197 | 786115 | 0.3634 | 0.3628 | 0.364 | 647 | 235 | 0.3632 | 0.3263 | 0.4018 |
| 2022 | >=80 | 2192592 | 875832 | 0.3995 | 0.3988 | 0.4001 | 584 | 207 | 0.3545 | 0.3159 | 0.395 |

**Figure 8:** Proportion of E10/E11 in patients with neuralgic amyotrophy as well as in all patients by year and age

**Table 18:** Proportion of E10/E11 in patients with neuralgic amyotrophy and in all patients by age. Values for age group 40-49 are based on years 2016,2017,2018,2020,2021 only as the data was incomplete for the others.


| Altersgruppe | Anzahl\_Versicherte\_gesamt | proportion\_E1011 | Anzahl\_Versicherte\_gesamt\_E10E11 | gesamt\_proportion\_E1011 | gesamt\_proportion\_E1011\_conf\_low | gesamt\_proportion\_E1011\_conf\_high | Anzahl\_Versicherte\_G54 | G54\_proportion\_E1011 | Anzahl\_Versicherte\_G54\_E10E11 | G54\_proportion\_E1011\_conf\_low | G54\_proportion\_E1011\_conf\_high |
| --- | --- | --- | --- | --- | --- | --- | --- | --- | --- | --- | --- |
| 40-49 | 3115805 | 0.06047 | 188404 | 0.06047 | 0.0602 | 0.0607 | 807 | 0.08674 | 70 | 0.0687 | 0.1088 |
| 50-59 | 3761947 | 0.1351 | 508422 | 0.1351 | 0.1348 | 0.1355 | 1255 | 0.1482 | 186 | 0.1292 | 0.1694 |
| 60-69 | 2981178 | 0.2596 | 773800 | 0.2596 | 0.2591 | 0.2601 | 994 | 0.2817 | 280 | 0.2541 | 0.311 |
| 70-79 | 2443457 | 0.371 | 906402 | 0.371 | 0.3703 | 0.3716 | 693 | 0.3737 | 259 | 0.3378 | 0.4111 |
| >=80 | 2132843 | 0.4034 | 860371 | 0.4034 | 0.4027 | 0.4041 | 527 | 0.3662 | 193 | 0.3253 | 0.4092 |

**Table 19:** Odds ratios with 95% confidence intervals and p values from contrast tests on a logistic regression model for diabetes. Values for age group 40-49 are based on years 2016,2017,2018,2020,2021 only as the data was incomplete for the others.


| Altersgruppe | contrast | OR | ci | p.value |
| --- | --- | --- | --- | --- |
| 40-49 | G54 / woG54 | 1.476 | [1.2; 1.9] | *< 0.01* |
| 50-59 | G54 / woG54 | 1.113 | [0.95; 1.3] | 0.18 |
| 60-69 | G54 / woG54 | 1.119 | [0.97; 1.3] | 0.11 |
| 70-79 | G54 / woG54 | 1.012 | [0.87; 1.2] | 0.88 |
| >=80 | G54 / woG54 | 0.8546 | [0.72; 1.0] | 0.08 |

**Figure 9:** Proportion of E10/E11 in patients with neuralgic amyotrophy and all patients by age. Values for age group 40-49 are based on years 2016,2017,2018,2020,2021 only as the data was incomplete for the others.

**Table 20:** Proportion of E10/E11 in patients with neuralgic amyotrophy and all patients. Note: Totals are calculated for all age groups 50-59, 60-69, 70-79, >=80 and for age group 40-49 in years 2016,2017,2018,2020,2021 as the data was incomplete for the others.


| Anzahl\_Versicherte\_gesamt | Anzahl\_Versicherte\_gesamt\_E10E11 | gesamt\_proportion\_E1011 | gesamt\_proportion\_E1011\_conf\_low | gesamt\_proportion\_E1011\_conf\_high | Anzahl\_Versicherte\_G54 | Anzahl\_Versicherte\_G54\_E10E11 | G54\_proportion\_E1011 | G54\_proportion\_E1011\_conf\_low | G54\_proportion\_E1011\_conf\_high |
| --- | --- | --- | --- | --- | --- | --- | --- | --- | --- |
| 14435230 | 3237399 | 0.2243 | 0.2241 | 0.2245 | 4276 | 988 | 0.2311 | 0.219 | 0.244 |

**Table 21:** Comparison of rates of E10/E11 between patients with and without neuralgic amyotrophy. Note: Totals are calculated for all age groups 50-59, 60-69, 70-79, >=80 and for age group 40-49 in years 2016,2017,2018,2020,2021 as the data was incomplete for the others.


| OR | 95% CI | p.value | method | alternative |
| --- | --- | --- | --- | --- |
| 1.039 | [0.967; 1.12] | 0.288 | Fisher’s Exact Test for Count Data | two.sided |

**Figure 10:** Proportion of E10/E11 in patients with neuralgic amyotrophy and all patients. Note: Totals are calculated for all age groups 50-59, 60-69, 70-79, >=80 and for age group 40-49 in years 2016,2017,2018,2020,2021 as the data was incomplete for the others.

# References

Lenth, Russell V. 2025. *Emmeans: Estimated Marginal Means, Aka Least-Squares Means*. https://rvlenth.github.io/emmeans/.

R Core Team. 2024. *R: A Language and Environment for Statistical Computing*. Vienna, Austria: R Foundation for Statistical Computing. https://www.R-project.org/.

# Session Info

```
R version 4.4.0 (2024-04-24)
Platform: x86_64-pc-linux-gnu
Running under: CentOS Linux 7 (Core)

Matrix products: default
BLAS/LAPACK: /opt/intel/oneapi/mkl/2023.0.0/lib/intel64/libmkl_gf_lp64.so.2;  LAPACK version 3.10.1

locale:
 [1] LC_CTYPE=en_US.utf8       LC_NUMERIC=C             
 [3] LC_TIME=en_US.utf8        LC_COLLATE=en_US.utf8    
 [5] LC_MONETARY=en_US.utf8    LC_MESSAGES=en_US.utf8   
 [7] LC_PAPER=en_US.utf8       LC_NAME=C                
 [9] LC_ADDRESS=C              LC_TELEPHONE=C           
[11] LC_MEASUREMENT=en_US.utf8 LC_IDENTIFICATION=C      

time zone: Europe/Berlin
tzcode source: system (glibc)

attached base packages:
[1] parallel  stats     graphics  grDevices datasets  utils     methods  
[8] base     

other attached packages:
 [1] doMC_1.3.8       iterators_1.0.14 foreach_1.5.2    ggeffects_2.2.0 
 [5] emmeans_1.10.7   ggplot2_3.5.1    broom_1.0.7      tidyr_1.3.1     
 [9] dplyr_1.1.4      readxl_1.4.3     descutils_0.1    remember_0.9    
[13] rmdcaptions_0.9  knitr_1.43       pander_0.6.5     rmarkdown_2.25  

loaded via a namespace (and not attached):
 [1] gtable_0.3.6       xfun_0.39          bslib_0.5.0        insight_1.0.1     
 [5] vctrs_0.6.5        tools_4.4.0        generics_0.1.3     tibble_3.2.1      
 [9] fansi_1.0.4        highr_0.10         RefManageR_1.4.0   pkgconfig_2.0.3   
[13] rematch_2.0.0      lifecycle_1.0.3    compiler_4.4.0     farver_2.1.2      
[17] stringr_1.5.1      git2r_0.32.0       munsell_0.5.1      codetools_0.2-19  
[21] htmltools_0.5.5    sass_0.4.7         yaml_2.3.7         pillar_1.9.0      
[25] jquerylib_0.1.4    cachem_1.0.8       zip_2.3.0          tidyselect_1.2.0  
[29] digest_0.6.31      mvtnorm_1.3-3      stringi_1.7.12     purrr_1.0.2       
[33] labeling_0.4.3     bibtex_0.5.1       fastmap_1.1.1      grid_4.4.0        
[37] colorspace_2.1-1   cli_3.6.1          magrittr_2.0.3     utf8_1.2.3        
[41] withr_2.5.0        scales_1.3.0       backports_1.4.1    lubridate_1.9.3   
[45] timechange_0.2.0   estimability_1.5.1 httr_1.4.6         cellranger_1.1.0  
[49] evaluate_0.21      rlang_1.1.4        Rcpp_1.0.13-1      glue_1.8.0        
[53] xml2_1.3.5         renv_1.0.3         jsonlite_1.8.9     R6_2.5.1          
[57] plyr_1.8.8
```
